# Nitrite Production by Nitrifying Bacteria in Urban Groundwater Used in a Chlorinated Public Bath System in Japan

**DOI:** 10.1264/jsme2.ME22040

**Published:** 2022-10-05

**Authors:** Yu Takahashi, Kento Ishii, Yukie Kikkawa, Kayo Horikiri, Satoshi Tsuneda

**Affiliations:** 1 Department of Life Science and Medical Bioscience, School of Advanced Science and Engineering, Waseda University, 2–2 Wakamatsu-cho, Shinjuku-ku, Tokyo, 162–8480, Japan; 2 Yokohama City Institute of Public Health, 2–7–1 Tomiokahigashi, Kanazawa-ku, Yokohama-shi, Kanagawa, 236–0051, Japan

**Keywords:** nitrification, chlorination, ammonia-oxidizing bacteria, nitrite-oxidizing bacteria, public bath

## Abstract

In contrast to pathogens, the effects of environmental microbes on the water quality in baths have not yet been examined in detail. We herein focused on a public bath in which groundwater was pumped up as bath water and disinfected by chlorination. Ammonia in groundwater is oxidized to nitrite, thereby reducing residual chlorine. A batch-culture test and bacterial community ana­lysis revealed that ammonia-oxidizing bacteria accumulated nitrite and had higher resistance to chlorination than nitrite-oxidizing bacteria. These results demonstrate that the difference in resistance to chlorination between ammonia-oxidizing and nitrite-oxidizing bacteria may lead to the accumulation of nitrite in baths using groundwater.

Public bath systems, such as spa pools, are common in some countries (Leoni *et al.*, 2018). However, public baths in which groundwater is pumped up and used as bath water are uncommon. In contrast, some public baths in Japan utilize groundwater for economic reasons, even though they are located in urban areas where tap water is available. Since these systems are not common worldwide, the effects of bacteria present in urban groundwater on the water quality of public baths have not yet been characterized in detail. Moreover, previous studies on microorganisms in bath systems have mainly focused on pathogenic bacteria, such as *Legionella* spp. (Kobayashi *et al.*, 2014; Leoni *et al.*, 2018). Therefore, we investigated the effects of environmental bacteria present in groundwater in a public bath system in an urban area of Japan. In this system, sodium hypochlorite (NaClO) was added to groundwater containing ammonium and the resulting chlorinated water was supplied to a public bath. However, the residual chlorine concentration in water decreased when it was transferred to the public bath. One of the reasons for this reduction is considered to have been nitrite accumulation induced by bacteria present in groundwater. Therefore, we attempted to identify bacteria involved in nitrite production in this public bath system.

All of the samples analyzed in the present study were collected from a public bath in Yokohama city, Japan. A schematic of the system is shown in Fig. 1. Groundwater was pumped from a confined aquifer. Combustible natural gases, such as methane, are present in aquifer groundwater (Tsunomori and Notsu, 2008; Matsushita *et al.*, 2020). These gases are removed from water using a gas-separation system (Tsunomori and Notsu, 2008). NaClO was added to the pumped water and stored in a concrete tank. A biofilm formed on the surface of the concrete tank. The tank was divided into two parts. The water stored in one tank was pumped into the other tank and was then slowly circulated. Water pumped from the tank was heated via heat exchange and sent to the public bath. The water samples analyzed in the present study were collected from the gas-separation system (Site X), water inlet (Site Y), and concrete water storage tank (Site Z) (Fig. 1). Wall biofilms were only collected from Site Z. Samples were stored at 4°C immediately after collection until the water quality ana­lysis, nitrification activity testing, and 16S rRNA gene sequencing were performed.

During the study period, groundwater chlorination conditions were revised to enhance disinfection efficiency. Table S1 lists chlorination conditions, the groundwater pumping rate, NaClO solution addition rate, and concentration of added NaClO solution. These measurements were used to calculate the final NaClO concentration in water supplied to the concrete tank. The NaClO solution addition rate increased from 5 to 30‍ ‍mL‍ ‍min^–1^ at groundwater pumping rates of 185 and 100 L‍ ‍min^–1^, respectively. The added NaClO solution concentration ranged between 61.6 and 124 Cl_2_ mg mL^–1^, corresponding to an increase in the theoretical NaClO concentration from 3.05 to 32.1 Cl_2_ mg L^–1^ (Table S1).

Water quality at the three sites was simultaneously characterized (see Supplemental Methods). Table S2 summarizes the characteristics of water quality. Water at all three sites had a medium temperature (21.2–33.9°C), low alkalinity (pH 8.0–8.3), and high total organic carbon load (74–100‍ ‍mg C L^–1^). Chloric acid was detected in water samples from Sites Y and Z (0.19–9.1‍ ‍mg L^–1^), which may have been produced by the reduction of NaClO. In contrast, free and combined chlorine concentrations at all sites were below the detection limit (1.0‍ ‍mg L^–1^). Water samples from the gas-separation system (Site X) had ammonium concentrations of 6.9–7.4‍ ‍mg L^–1^ (Table S2). Ammonium reacts with NaClO and converts it to chloramine, a type of combined chlorine (Vikesland *et al.*, 2001). Other substances, such as ferrous iron and natural organic matter, have been shown to contribute to the decay of chloramine (Curling *et al.*, 2022). These substances may have decreased the residual chlorine concentration. Furthermore, nitrification may have occurred in the concrete tank. Although water samples from the gas-separation system (Site X) contained high concentrations of ammonium, nitrite and nitrate concentrations were both below 0.1‍ ‍mg L^–1^ (Table S2). In contrast, nitrite and nitrate concentrations were higher than 0.1‍ ‍mg L^–1^ in water samples from the water inlet (Site Y) and the water storage tank (Site Z). These results suggest that ammonium in the water was oxidized by nitrification when it was stored in the concrete water tank. However, nitrite and nitrate concentrations at the water inlet (Site Y) gradually decreased (Table S2), whereas the amount of NaClO supplied to the tank increased (Table S1). Specifically, the nitrate concentration range at Site Y decreased from 2.3–4.9‍ ‍mg L^–1^ between January 14 and March 16, 2020 to 0.14–0.17‍ ‍mg L^–1^ between September 8 and October 20, 2020. Therefore, increased NaClO supplied to the concrete tank may have inhibited nitrification activity despite the low residual chlorine concentrations detected in the tank.

The inhibitory effects of NaClO on nitrification activity were evaluated in a laboratory (see Supplemental Methods for details on the experiment). Briefly, water samples from Sites X and Y were mixed and separated into six test tubes supplemented with NaClO (0, 1.2, 2.3, 3.5, 4.6, and 5.8‍ ‍mg‍ ‍L^–1^, final concentration). Approximately 5‍ ‍mg L^–1^ ammonium was detected in all test tubes at the beginning of the test, but was completely consumed after the addition of NaClO (Fig. 2A). Although ammonium consumption was delayed by 4.6 and 5.8‍ ‍mg L^–1^ NaClO, its concentration reached zero within 6 days. Moreover, the concentration of nitrite increased to approximately 6‍ ‍mg L^–1^ under all conditions (Fig. 2B). The maximum nitrite concentration (approximately 6‍ ‍mg L^–1^) was higher than the initial ammonium concentration (approximately 5‍ ‍mg L^–1^) (Fig. 2A and B). This may be due to nitrogen mineralization, which converts organic nitrogen to ammonium (Rusydi *et al.*, 2021). This hypothesis is supported by the result showing that ammonium concentrations increased to approximately 6‍ ‍mg L^–1^ at NaClO concentrations of 4.6 and 5.8‍ ‍mg L^–1^ (Fig. 2A). Furthermore, the nitrite concentration decreased (Fig. 2B) and nitrate concentration increased (Fig. 2C) 120‍ ‍h after the addition of 0, 1.2, and 2.3‍ ‍mg L^–1^ NaClO. In conclusion, the ammonia-oxidizing activity of the groundwater biomass was still detected after the treatment with NaClO at concentrations of 3.5, 4.6, and 5.8‍ ‍mg L^–1^, whereas nitrite oxidation was completely inhibited under these conditions. This result suggests that NaClO exerted stronger inhibitory effects on nitrite oxidation than on ammonia oxidation. Likewise, the nitrite oxidation rate in the concrete tank may have been slower than the ammonia oxidation rate. This difference between the two reaction rates may have resulted in the accumulation of nitrite at the water inlet (Site Y) (Table S2). Nitrite reacts with residual chlorine and decreases the disinfection effects of chlorination (Cruz *et al.*, 2020). Therefore, nitrite accumulation in a public bath system may also decrease the effects of chlorination.

Bacterial community compositions were analyzed to clarify the taxonomy of nitrifiers in the public bath system. Water samples were collected from the gas-separation system and water inlet (Sites X and Y) on February 26, 2020. A wall biofilm sample was collected from the concrete tank (Site Z) on October 20, 2020. The genomic DNA extracted from these samples was analyzed using 16S rRNA gene amplicon sequencing (see Supplemental Methods). Sequencing data were deposited in the DNA Databank of Japan (DDBJ), EMBL Nucleotide Sequence Database, and the National Center for Biotechnology Information (NCBI) database under project code PRJDB13264, with raw sequence data under accession number DRA013744. *Nitrosomonadaceae* and *Nitrospiraceae*, known as ammonia-oxidizing bacteria (AOB) and nitrite-oxidizing bacteria (NOB), respectively, were detected in this system. In water from the gas-separation system, the relative abundance of *Nitrosomonadaceae* and *Nitrospiraceae* accounted for 13.5 and 14.0%, respectively (Fig. 3A). Despite their high abundance in water from the gas-separation system, these two families were not detected in samples from the water inlet. The addition of NaClO may have decreased the abundance of the nitrifiers. However, the wall biofilm from the concrete tank was dominated by *Nitrosomonadaceae* (39.8%). Therefore, the ammonium present in groundwater may have been oxidized by AOB in the biofilm on the concrete tank surface as well as planktonic AOB in the water. Moreover, the wall biofilm was collected from the concrete tank on October 20, 2020, after the addition of NaClO had increased (Table S1) and nitrate production had decreased (Table S2). Consequently, nitrifiers in the water tank may have been more abundant on February 26, 2020, when samples were collected from the gas-separation system and water inlet, than on October 20, 2020.

In contrast to *Nitrosomonadaceae*, the abundance of *Nitrospiraceae* in the water tank was only 2.7% (Fig. 3A). This difference in the abundance of *Nitrosomonadaceae* and *Nitrospiraceae* may be attributed to differences in their tolerance to chlorination. *Nitrospiraceae* is one of the dominant taxa in non-chlorinated drinking water wells (Karwautz and Lueders, 2014) and groundwater (Geesink *et al.*, 2022). Moreover, a study on drinking water distribution systems (DWDSs) supplied with chloramine (a type of residual chlorine) reported a low abundance of *Nitrospira*, but relatively high abundance of *Nitrosomonas* in areas with high chloramine concentrations (Cruz *et al.*, 2020). Therefore, a low abundance of *Nitrospiraceae* may be common among systems treated with residual chlorine.

In addition to the nitrifiers described above, *Methylophilaceae* and *Methylomonaceae* were among the major families identified in the bath system (Fig. 3A). *Methylophilaceae* are methylotrophic (Chistoserdova, 2015), whereas *Methylomonaceae* are methanotrophic (Ho *et al.*, 2020). Furthermore, *Enterobacteriaceae* was detected in water from the gas-separation system and water inlet (Fig. 3A). This family includes *Salmonella* and *Escherichia coli* O157, which cause groundwater-related diseases (Murphy *et al.*, 2017). No members of the *Legionellaceae* family, including *Legionella*, which causes Legionnaires’ disease (Leoni *et al.*, 2018), were detected in the present study. Moreover, nitrogen cycle-related bacteria other than nitrifiers were identified. *Candidatus* Brocadiaceae accounted for 0.29% of the bacterial community in water from the gas-separation system (Table S3). This family includes anaerobic ammonia-oxidizing (anammox) bacteria (Kumwimba *et al.*, 2020). *Pseudomonadaceae* (Fig. 3A) and *Moraxellaceae* (Table S3) were detected in water from the gas separation system and water inlet. These families include *Pseudomonas* spp. and *Acinetobacter* spp., which are aerobic denitrifiers (Ji *et al.*, 2015). Although these taxa were not abundant in the bath system examined, they may have contributed to the consumption of ammonium, nitrite, and nitrate via anammox or denitrification.

Fig. 3B shows the relative abundance of amplicon sequence variants (ASVs) classified as *Nitrosomonadaceae* and *Nitrospiraceae*. Three *Nitrosomonadaceae* ASVs, YKHM01, YKHM02, and YKHM03, were identified. YKHM02 was the most abundant AOB in water from the gas-separation system, accounting for 12.9% of the whole bacterial community. Contrary, on the biofilm on the wall of the concrete tank, YKHM01 (35.9%) and YKHM03 (4.0%) were the most abundant AOB. In contrast to AOB, the most abundant NOB in samples from the gas-separation system and water inlet were similar (Fig. 3B). YKHM04 of *Nitrospiraceae* accounted for 13.3 and 2.7% in samples from the gas-separation system and water inlet, respectively.

The ASVs of *Nitrosomonadaceae* and *Nitrospiraceae* in the present study were analyzed to identify the phylogeny of nitrifiers (Supplemental Methods). The nucleotide sequences of these ASVs were deposited in the DDBJ, EMBL, and NCBI databases under accession numbers LC703036–LC703040. All three ASVs of *Nitrosomonadaceae* were classified into *Nitrosomonas* Cluster 6 (Fig. 4A). Cluster 6 is divided into Clusters 6a and 6b (Prosser *et al.*, 2014). However, we did not identify the cluster subtype to which YKHM01 and YKHM03 belonged because the ASVs obtained in the present study were only 335 nucleotides long. Despite the difficulties associated with conducting a detailed phylogenetic ana­lysis of subgroups, the major AOB in water from the gas-separation system and water inlet (YKHM02 and YKHM01, respectively) occupied distinct phylogenetic positions in Cluster 6. Similarly, clones of *Nitrosomonas* Cluster 6a have been detected as the major AOB in several DWDSs supplied with chloramine (Regan *et al.*, 2003); therefore, the *Nitrosomonas* species detected in the present study are common AOB in chlorinated systems.

The ASVs of *Nitrospiraceae* detected in this study were classified as *Nitrospira* lineage I (Fig. 4B). Although this genus contains complete ammonia-oxidizing (comammox) *Nitrospira*, which oxidizes ammonia and nitrite in a single organism, all previously reported comammox bacteria have been classified as lineage II (Koch *et al.*, 2019). Therefore, the ASVs detected in the present study were NOB, only oxidizing nitrite to nitrate. *Nitrospira* species are the main NOB in some chloraminated DWDSs (Regan *et al.*, 2003; Cruz *et al.*, 2020). However, other NOB, such as *Ca.* Nitrotoga and *Nitrobacter*, have also been detected in chloraminated DWDSs (Waak *et al.*, 2019). Further studies are required to elucidate the relationship between NOB resistance to residual chlorine and their phylogeny.

Based on the results of the nitrification activity test (Fig. 2) and bacterial community ana­lysis (Fig. 3), the NOB (*Nitrospira* lineage I) detected in the present study were more sensitive to chlorination than AOB (*Nitrosomonas* cluster 6). Similarly, in a previous study on chloraminated DWDSs, *Nitrospira* was abundant in an environment with a low residual chlorine concentration, whereas *Nitrosomonas* was abundant in environments with high chlorine concentrations (Cruz *et al.*, 2020). This difference in residual chlorine resistance between *Nitrosomonas* and *Nitrospira* may lead to the accumulation of nitrite. Nitrite reacts with monochloramine to form ammonium, nitrate, and chloride ions (Cruz *et al.*, 2020). AOB promote residual chlorine decomposition through ammonia oxidation and subsequent nitrite formation (Keshvardoust *et al.*, 2020). Nitrification in DWDSs disrupts the maintenance of a constant amount of residual chlorine (Zhang *et al.*, 2009). Therefore, AOB activity needs to be inhibited in DWDSs and public bath systems in order to prevent the accumulation of nitrite and microbial contamination. Nitrification in waterworks is active in environments with chloramine concentrations of 2–3‍ ‍mg L^–1^ (Wilczak *et al.*, 1996). Research on chlorinated waterworks will provide important information for the selection of the residual chlorine concentration needed to inhibit nitrite accumulation in Japanese public bath systems. Moreover, the physiological characterization of AOB and NOB will help to explain differences in the chloramine resistance of *Nitrosomonas* and *Nitrospira*. The chloramine tolerance of the AOB isolate *Nitrosomonas europaea* has been investigated (Oldenburg *et al.*, 2002) and was found to be more resistant to chloramine than *E. coli* O157:H7 (Chauret *et al.*, 2008). However, to the best of our knowledge, similar studies on NOB have yet to be conducted. Physiological experiments on NOB isolates are also needed to compare the chloramine resistance of NOB and AOB.

In conclusion, the present study revealed that nitrifying bacteria contributed to a decrease in residual chlorine in a public bath system through the accumulation of nitrite. In this system, NaClO was supplied to groundwater in order to inhibit pathogens, such as *Legionella*. However, AOB in groundwater accumulated nitrite, which may have decreased the bactericidal activity of residual chlorine. In contrast, NOB did not completely oxidize nitrite because it was less resistant than AOB to residual chlorine. Furthermore, the nitrifier ASVs detected in the bath system were phylogenetically related to the clones detected in waterworks supplied with residual chlorine. Therefore, further studies on chlorination in waterworks, such as DWDSs, may provide insights that will be important for the appropriate operation of chlorinated Japanese public bath systems.

## Citation

Takahashi, Y., Ishii, K., Kikkawa, Y., Horikiri, K., and Tsuneda, S. (2022) Nitrite Production by Nitrifying Bacteria in Urban Groundwater Used in a Chlorinated Public Bath System in Japan. *Microbes Environ ***37**: ME22040.

https://doi.org/10.1264/jsme2.ME22040

## Supplementary Material

Supplementary Material

## Figures and Tables

**Fig. 1.**
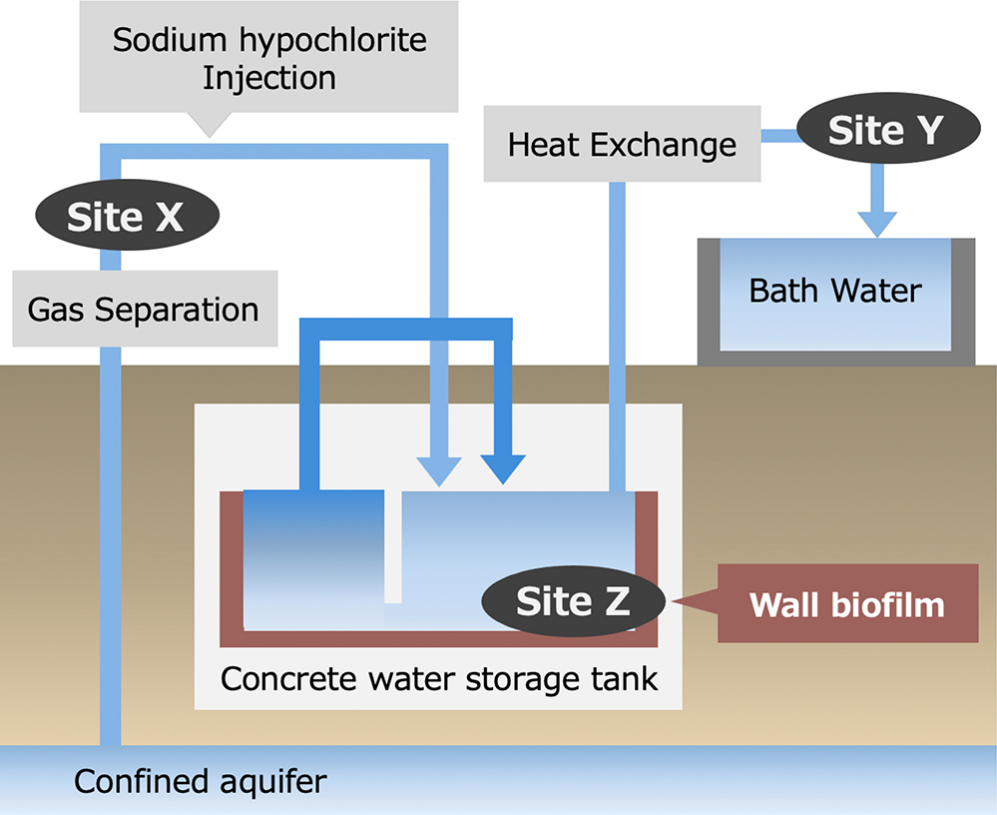
Schematic diagram of the studied urban public bath system. Arrows indicate water distribution. Black ellipses represent sampling sites. The concrete water storage tank surface (Site Z) was covered with a biofilm. The concrete tank was divided into two sections. Stored water was pumped between the two sections to slowly circulate the water.

**Fig. 2.**
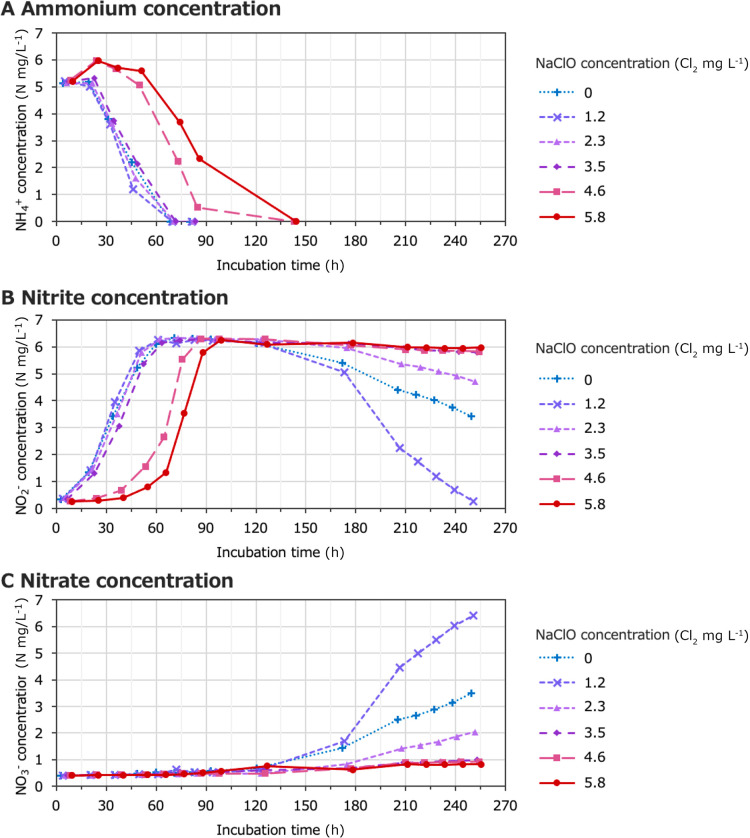
The nitrification potential of sampled water from an urban public bath system. Groundwater collected from the gas-separation system (Site X) and water inlet (Site Y) (see Fig. 1) were mixed and supplemented with sodium hypochlorite (NaClO). The concentrations of (A) ammonium, (B) nitrite, and (C) nitrate were measured.

**Fig. 3.**
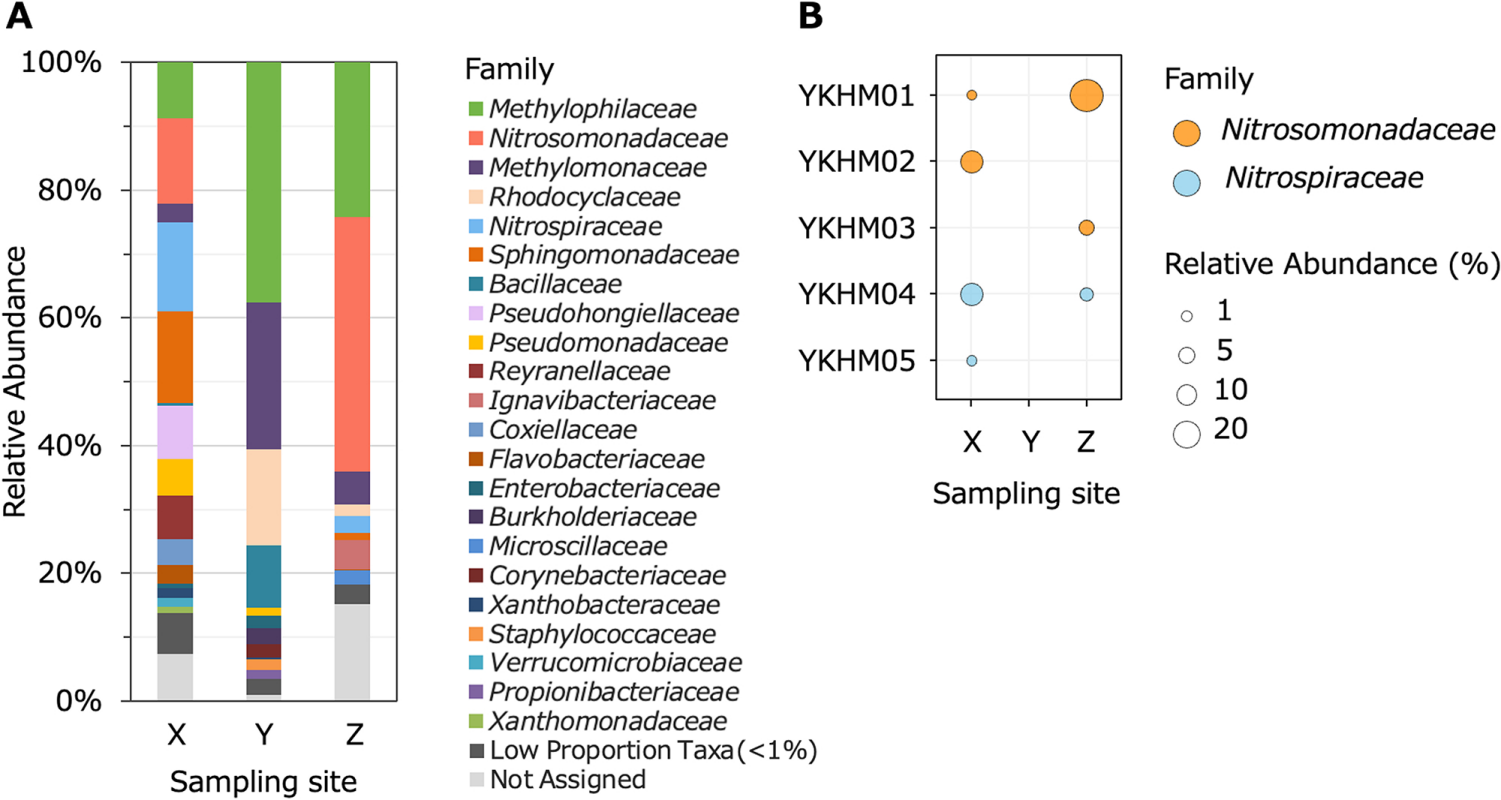
The bacterial community based on the V7–V8 region of 16S rRNA gene sequences from the biomass in an urban public bath system. Sampling sites were the gas-separation system (Site X), water inlet (Site Y), and concrete water storage tank (Site Z) (see Fig. 1). (A) The relative proportions of families. The taxa accounting for <1% were grouped into “Low Proportion Taxa”. The taxa unable to be annotated were grouped into “Not Assigned”. (B) The relative abundance of amplicon sequencing variants of nitrifiers. The plot size indicates relative abundance to the whole bacterial community. The plot color corresponds to the families (orange: *Nitrosomonadaceae*, cyan: *Nitrospiraceae*).

**Fig. 4.**
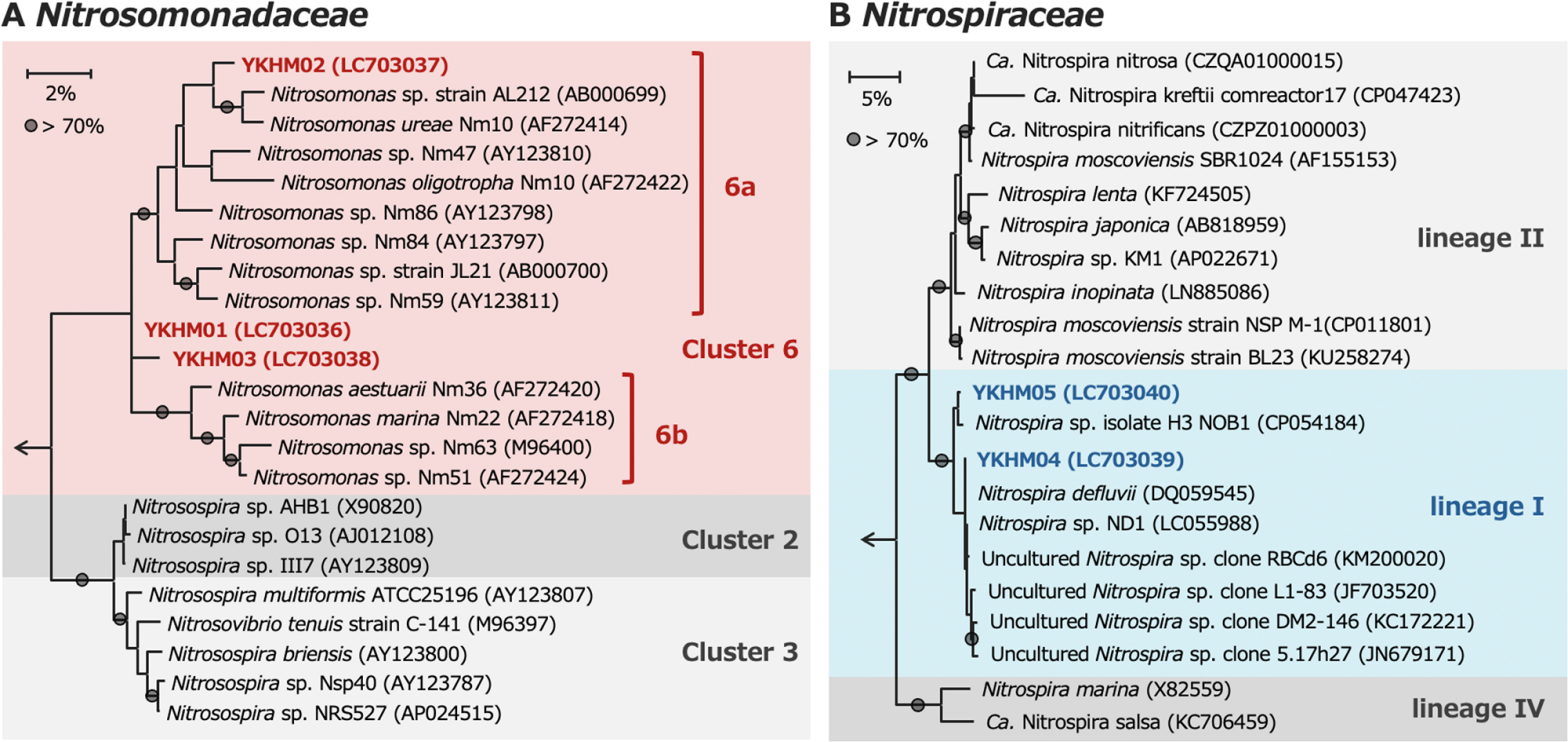
The phylogeny of nitrifying bacteria detected in an urban public bath system. Maximum-likelihood phylogenetic trees were reconstructed based on the 16S rRNA gene sequences of (A) *Nitrosomonadaceae* and (B) *Nitrospiraceae*. Bootstrap values were calculated based on 1,000 iterations. The letters in parentheses indicate accession numbers. The sequences obtained in the present study are indicated in bold. Color shades indicate clusters or lineages. Arrows indicate outgroups.
