# Supplementary material for: Nitrite Production by Nitrifying Bacteria in Urban Groundwater Used in a Chlorinated Public Bath System in Japan
Source: Microbes Environ. 2022 Oct 5;37(4):ME22040. doi: 10.1264/jsme2.ME22040 (PMC9763042; doi:10.1264/jsme2.ME22040)
Supplement: Supplementary file 1 — Supplementary Material [file 37_22040_s1.pdf]

Supplemental Tables

Table S1. The condition of sodium hypochlorite (NaClO) treatment.

Date (Day/Month/Year)	Groundwater pumping rate[a] [L/min]	NaClO solution addition rate[b] [mL/min]	Added NaClO solution concentration[c] [mg-Cl_2/mL]	Theoretical final NaClO concentration[d] [mg-Cl_2/L]
14/01/2020	185	5	113	3.05
26/02/2020	185	20	124	13.4
16/03/2020	185	30	118	19.1
08/09/2020	100	30	61.6	18.5
14/10/2020	100	30	107	32.1

NaClO solution was added to the pumped groundwater. The final NaClO concentration in water (d) was theoretically calculated based on a, b, and c (d=b×c/a).

Table S2. Water quality characterization.

Site[a]	Sampling date (Day/Month/Year)	Parameter [unit]								
		Temperature [°C]	pH	TOC[b] [mg-C/L]	Chloric acid [mg-Cl_2/L]	Free Chlorine [mg-Cl_2/L]	Combined Chlorine [mg-Cl_2/L]	Ammonium [mg-N/L]	Nitrite [mg-N/L]	Nitrate [mg-N/L]
X	14/01/2020	27.9	8.1	86	<0.06	<1.0	<1.0	7.3	<0.004	<0.1
	26/02/2020	N.A.	8.1	76	<0.06	<1.0	<1.0	6.9	0.0061	<0.1
	16/03/2020	23.4	8.2	79	<0.06	<1.0	<1.0	7.0	<0.004	<0.1
	08/09/2020	21.2	8.2	77	<0.06	<1.0	<1.0	7.4	<0.004	<0.1
Y	14/01/2020	33.9	8.0	100	0.19	<1.0	<1.0	0.75	1.33	4.1
	26/02/2020	N.A.	8.0	79	0.94	<1.0	<1.0	0.50	1.45	2.3
	16/03/2020	28.5	8.0	83	0.98	<1.0	<1.0	<0.1	1.33	4.9
	08/09/2020	30	8.3	74	8.1	<1.0	<1.0	6.6	0.207	0.14
	14/10/2020	N.A.	8.3	84	2.2	<1.0	<1.0	5.8	0.227	0.17
Z	08/09/2020	21.5	8.3	90	9.1	<1.0	<1.0	6.7	0.138	0.11
	20/10/2020	N.A.	8.2	N.A.	6.8	<1.0	<1.0	4.2	0.110	0.15

N.A.: Not analyzed. Inequality signs (<: less than) indicate the values are under detection limits.

[a]Sampling sites are coordinate with the locations shown in Figure 1.

[b]TOC: Total organic carbon.

Table S3. The relative abundances of the taxa possessing less than 1% of the total bacterial communities.

Family	Site X	Site Y	Site Z
Beijerinckiaceae	0.62%	0.38%	0.00%
Xanthomonadaceae	0.98%	0.00%	0.00%
Parachlamydiaceae	0.92%	0.00%	0.00%
Solimonadaceae	0.92%	0.00%	0.00%
Rhizobiaceae	0.00%	0.00%	0.87%
Chitinophagaceae	0.85%	0.00%	0.00%
Phycisphaeraceae	0.00%	0.00%	0.81%
Opitutaceae	0.00%	0.00%	0.74%
Moraxellaceae	0.33%	0.38%	0.00%
Mycobacteriaceae	0.69%	0.00%	0.00%
Nocardiaceae	0.59%	0.00%	0.00%
Unclassified family of *Flavobacteriales*	0.52%	0.00%	0.00%
Solibacteraceae	0.00%	0.46%	0.00%
Gemmatimonadaceae	0.00%	0.00%	0.40%
Alcanivoracaceae	0.00%	0.38%	0.00%
Marinobacteraceae	0.00%	0.34%	0.00%
Rickettsiaceae	0.33%	0.00%	0.00%
Brocadiaceae	0.29%	0.00%	0.00%
Victivallaceae	0.00%	0.00%	0.27%
Unclassified family of *Ignavibacteriales*	0.00%	0.27%	0.00%
Hyphomicrobiaceae	0.23%	0.00%	0.00%
Spirochaetaceae	0.00%	0.19%	0.00%
Gemmataceae	0.00%	0.11%	0.00%
Blastocatellaceae	0.10%	0.00%	0.00%

Supplemental Methods

Water quality characterization

Ammonium, nitrite, nitrate, and chloric acid concentrations were measured using a Dionex ICS-1600 ion chromatograph (Thermo Fisher Scientific, Waltham, MA, USA). Only nitrite concentration was measured after adding ethylenediamine to the samples. Residual (free and combined) chlorine concentrations were measured using the N, N-diethyl-p-phenylenediamine (DPD)-based colorimetric method and the absorbance was measured using a V-660 UV-VIS spectrophotometer (JASCO Corporation, Tokyo, Japan). Total organic carbon concentrations were measured using a multi N/C UV HS (Analytik Jena, Jena, Germany).

Nitrification activity test

Water samples were collected from sites X and Y on February 26, 2020. Water (540 mL from site X and 60 mL from site Y) were mixed and dispensed into six color comparison tubes (100 mL water per tube). Then, 0, 2, 4, 6, 8, or 10 μL 5.8% w/v NaClO solution was added to each tube (final NaClO concentrations: 0, 1.2, 2.3, 3.5, 4.6, or 5.8 mg-Cl_2/L, respectively) and incubated at 30 °C. Ammonium, nitrite, and nitrate concentrations were measured. Ammonium was not added as the substrate for nitrification.

Bacterial community analysis based on 16S rRNA gene amplicon sequencing

Water samples were collected from sites X and Y on February 26, 2020. A wall biofilm sample was collected from site Z on October 20, 2020. The genomic DNA in these samples was extracted using the FastDNA Spin Kit for Soil (MP Biomedicals LLC, Irvine, CA, USA) and stored at −20 °C until use for PCR. The V7 and V8 regions of the bacterial 16S rRNA genes were amplified with Ex Taq (Takara Bio Inc., Shiga, Japan) and a PCR primer set: 1055f (Ferris et al., 1996) and 1392r (Lane et al., 1991). The sequences of primers were 5’-

ATGGCTGTCGTCAGCT-3’ (1055f) and 5’-ACGGGCGGTGTGTAC-3’ (1392r), respectively. The adapter sequences were attached to primers. The PCR conditions were as follows: 95 °C for 5 min; 30 cycles of 95 °C for 30 s, 52 °C for 30 s, 72 °C for 30 s; followed by 72 °C for 4 min. The PCR product was purified using a FastGene Gel/PCR Extraction Kit (Nippon Genetics Co., Ltd., Tokyo, Japan). Template amplification and amplicon enrichment were performed using the Ion One-Touch™ 2 system with Ion PGM™ Hi-Q™ View OT2 Kit-400 (Thermo Fisher Scientific, Waltham, MA, USA). An Ion Personal Genome Machine (Ion PGM™) system with the Ion PGM™ Hi-Q™ View Sequencing Kit and Ion 318™ Chip Kit v2 (Thermo Fisher Scientific, Waltham, MA, USA) was used for sequencing. Sequence data was analyzed using DADA2 (Divisive Amplicon Denoising Algorithm 2) version 3.10 (Callahan et al., 2016). Quality checks, chimera removal, and trimming were performed using the filterAndTrim function. The reads were truncated after 350 bases and 15 nucleotides from the start of each read were trimmed. Reads including Ns (maxN=0), high expected errors (maxEE=2), and low quality scores (truncQ=2) were excluded according to the developer’s suggestions (https://benjjneb.github.io/dada2/faq.html#can-i-use-dada2-with-my-454-or-ion-torrent-data). The amplicon sequence variants (ASVs) were acquired and their taxonomy was assigned using SILVA database version 132 (Quast et al., 2013).

Phylogenetic analysis

The sequences related to the ASVs acquired in this study were searched in the NCBI nucleotide database (NCBI Resource Coordinators, 2016). Phylogenetic trees based on the 16S rRNA gene sequences were constructed using MEGA X software (Kumar et al., 2018) according to the maximum-likelihood method and Jukes–Cantor model (Jukes and Cantor, 1969) with 1,000 bootstraps. The outgroup in Fig. 4A comprises

Nitrosococcus halophilus Nc4 (accession number: AF287298) and *N. watsonii* strain C-113 (NR_074791). The outgroup in Fig. 4B comprises *Leptospirillum ferrooxidans* strain Z2 (AH001683) and *L. ferriphilum* strain P3a (NR_028818).

References

Callahan, B.J., McMurdie, P.J., Rosen, M.J., Han, A.W., Johnson, A.J., and Holmes, S.P. (2016) DADA2: High-resolution sample inference from Illumina amplicon data. *Nat Methods* **13**: 581-583.

Ferris, M.J., Muyzer, G., and Ward, D.M. (1996) Denaturing gradient gel electrophoresis profiles of 16S rRNA-defined populations inhabiting a hot spring microbial mat community. *Appl Environ Microbiol* **62**: 340-346.

Jukes, T.H. and Cantor, C.R. (1969) Evolution of Protein Molecules. In: Munro, H.N., Ed., Mammalian Protein Metabolism, Academic Press, New York, pp. 21-132.

Kumar, S., Stecher, G., Li, M., Knyaz, C., and Tamura, K. (2018) MEGA X: Molecular Evolutionary Genetics Analysis across Computing Platforms. *Mol Biol Evol* **35**: 1547-1549.

Lane, D.J. (1991) 16S/23S rRNA Sequencing. In Nucleic Acid Techniques in Bacterial Systematic. New York, John Wiley and Sons, pp. 115-175.

NCBI Resource Coordinators (2016) Database resources of the National Center for Biotechnology Information. *Nucleic Acids Res* **44**: D7-D19.

Quast, C., Pruesse, E., Yilmaz, P., Gerken, J., Schweer, T., Yarza, P., Peplies, J., and Glöckner, F.O. (2013) The SILVA ribosomal RNA gene database project: improved data processing and web-based tools. *Nucleic Acids Res* **41**: D590-D596.
